# Supplementary material for: Transcriptome Analysis Reveals Vimentin-Induced Disruption of Cell–Cell Associations Augments Breast Cancer Cell Migration
Source: Cells. 2022 Dec 13;11(24):4035. doi: 10.3390/cells11244035 (PMC9776984; doi:10.3390/cells11244035)
Supplement: Supplementary file 1 [file cells-11-04035-s001.zip › DEGs and enrichment analysis.zip!/DEGs and enrichment analysis/WT_VIMvsVector KEGG/GSEA/WT_VIM/DNA_REPLICATION/DNA_REPLICATION(HSA03030).html]

Details for gene set DNA\_REPLICATION(HSA03030)[GSEA]

|  || Dataset | fpkm.sample |
| Phenotype | sample.cls |
| Upregulated in class | WT\_VIM |
| GeneSet | DNA\_REPLICATION(HSA03030) |
| Enrichment Score (ES) | 0.7363942 |
| Normalized Enrichment Score (NES) | 1.276597 |
| Nominal p-value | 0.0 |
| FDR q-value | 0.1649999 |
| FWER p-Value | 0.165 |
Table: GSEA Results Summary

  

Fig 1: Enrichment plot: DNA\_REPLICATION(HSA03030)      
 Profile of the Running ES Score & Positions of GeneSet Members on the Rank Ordered List

  

| PROBE | DESCRIPTION (from dataset) | GENE SYMBOL | GENE\_TITLE | RANK IN GENE LIST | RANK METRIC SCORE | RUNNING ES | CORE ENRICHMENT || 1 | ENSG00000283544 | ABBA01000935.2 |  |  | 869 | Infinity | -0.0146 | Yes |
| 2 | ENSG00000073111 | MCM2 |  |  | 2938 | 5.216 | 0.0093 | Yes |
| 3 | ENSG00000104889 | RNASEH2A |  |  | 3022 | 4.780 | 0.0618 | Yes |
| 4 | ENSG00000100297 | MCM5 |  |  | 3043 | 4.701 | 0.1145 | Yes |
| 5 | ENSG00000166508 | MCM7 |  |  | 3213 | 4.212 | 0.1592 | Yes |
| 6 | ENSG00000062822 | POLD1 |  |  | 3291 | 3.989 | 0.2029 | Yes |
| 7 | ENSG00000168496 | FEN1 |  |  | 3538 | 3.632 | 0.2397 | Yes |
| 8 | ENSG00000106628 | POLD2 |  |  | 3557 | 3.585 | 0.2799 | Yes |
| 9 | ENSG00000198056 | PRIM1 |  |  | 3560 | 3.578 | 0.3203 | Yes |
| 10 | ENSG00000049541 | RFC2 |  |  | 3833 | 3.234 | 0.3522 | Yes |
| 11 | ENSG00000105486 | LIG1 |  |  | 4197 | 2.863 | 0.3783 | Yes |
| 12 | ENSG00000014138 | POLA2 |  |  | 4218 | 2.849 | 0.4101 | Yes |
| 13 | ENSG00000112118 | MCM3 |  |  | 4242 | 2.827 | 0.4417 | Yes |
| 14 | ENSG00000231458 | RNASEH1P2 |  |  | 4557 | 2.637 | 0.4661 | Yes |
| 15 | ENSG00000132646 | PCNA |  |  | 4836 | 2.473 | 0.4893 | Yes |
| 16 | ENSG00000177084 | POLE |  |  | 5060 | 2.371 | 0.5123 | Yes |
| 17 | ENSG00000104738 | MCM4 |  |  | 5576 | 2.171 | 0.5281 | Yes |
| 18 | ENSG00000100479 | POLE2 |  |  | 5908 | 2.060 | 0.5457 | Yes |
| 19 | ENSG00000133119 | RFC3 |  |  | 6033 | 2.025 | 0.5665 | Yes |
| 20 | ENSG00000076003 | MCM6 |  |  | 6061 | 2.015 | 0.5888 | Yes |
| 21 | ENSG00000117748 | RPA2 |  |  | 7021 | 1.813 | 0.5930 | Yes |
| 22 | ENSG00000106399 | RPA3 |  |  | 7836 | 1.651 | 0.5979 | Yes |
| 23 | ENSG00000172922 | RNASEH2C |  |  | 8057 | 1.615 | 0.6124 | Yes |
| 24 | ENSG00000111445 | RFC5 |  |  | 8152 | 1.601 | 0.6288 | Yes |
| 25 | ENSG00000132383 | RPA1 |  |  | 8219 | 1.591 | 0.6457 | Yes |
| 26 | ENSG00000163918 | RFC4 |  |  | 8301 | 1.578 | 0.6621 | Yes |
| 27 | ENSG00000269534 | AC011466.3 |  |  | 8453 | 1.552 | 0.6771 | Yes |
| 28 | ENSG00000101868 | POLA1 |  |  | 8951 | 1.488 | 0.6855 | Yes |
| 29 | ENSG00000138346 | DNA2 |  |  | 9058 | 1.472 | 0.7003 | Yes |
| 30 | ENSG00000148229 | POLE3 |  |  | 9318 | 1.440 | 0.7122 | Yes |
| 31 | ENSG00000115350 | POLE4 |  |  | 9648 | 1.399 | 0.7224 | Yes |
| 32 | ENSG00000077514 | POLD3 |  |  | 9747 | 1.389 | 0.7364 | Yes |
| 33 | ENSG00000204086 | RPA4 |  |  | 11238 | 1.244 | 0.7252 | No |
| 34 | ENSG00000171865 | RNASEH1 |  |  | 13022 | 1.100 | 0.7074 | No |
| 35 | ENSG00000136104 | RNASEH2B |  |  | 13417 | 1.070 | 0.7129 | No |
| 36 | ENSG00000146143 | PRIM2 |  |  | 14384 | 0.996 | 0.7077 | No |
| 37 | ENSG00000215796 | AL512637.1 |  |  | 14502 | 0.996 | 0.7170 | No |
| 38 | ENSG00000035928 | RFC1 |  |  | 14751 | 0.986 | 0.7239 | No |
| 39 | ENSG00000106028 | SSBP1 |  |  | 16461 | 0.890 | 0.7050 | No |
| 40 | ENSG00000175482 | POLD4 |  |  | 17979 | 0.800 | 0.6884 | No |
| 41 | ENSG00000284491 | THSD8 |  |  | 24368 | 0.410 | 0.5849 | No |
| 42 | ENSG00000277873 | AC131159.2 |  |  | 27133 | 0.080 | 0.5390 | No |
| 43 | ENSG00000258392 | RPA2P1 |  |  | 27356 | 0.000 | 0.5352 | No |
| 44 | ENSG00000216475 | AL024474.1 |  |  | 30460 | 0.000 | 0.4827 | No |
| 45 | ENSG00000233526 | RFC5P1 |  |  | 33745 | NaN | 0.4272 | No |
| 46 | ENSG00000224529 | AC093698.2 |  |  | 33897 | NaN | 0.4248 | No |
| 47 | ENSG00000215873 | FEN1P1 |  |  | 37119 | NaN | 0.3704 | No |
| 48 | ENSG00000265790 | RNASEH1P1 |  |  | 39430 | NaN | 0.3314 | No |
| 49 | ENSG00000236158 | RFC3P1 |  |  | 45281 | NaN | 0.2325 | No |
| 50 | ENSG00000237936 | AL353093.1 |  |  | 46045 | NaN | 0.2197 | No |
| 51 | ENSG00000256514 | AP003419.1 |  |  | 46088 | NaN | 0.2191 | No |
| 52 | ENSG00000213730 | POLD2P1 |  |  | 46754 | NaN | 0.2079 | No |
| 53 | ENSG00000255547 | RPA2P3 |  |  | 49116 | NaN | 0.1681 | No |
| 54 | ENSG00000232482 | AL357134.1 |  |  | 49526 | NaN | 0.1612 | No |
| 55 | ENSG00000283453 | AC244258.1 |  |  | 51447 | NaN | 0.1289 | No |
| 56 | ENSG00000249065 | PCNAP1 |  |  | 52056 | NaN | 0.1187 | No |
| 57 | ENSG00000233455 | AL035414.1 |  |  | 52247 | NaN | 0.1156 | No |
| 58 | ENSG00000218261 | AL591034.1 |  |  | 52536 | NaN | 0.1108 | No |
| 59 | ENSG00000254544 | PCNAP4 |  |  | 52840 | NaN | 0.1058 | No |
| 60 | ENSG00000214009 | PCNAP3 |  |  | 54078 | NaN | 0.0850 | No |
| 61 | ENSG00000226466 | RPA2P2 |  |  | 56081 | NaN | 0.0512 | No |
| 62 | ENSG00000228721 | AC016909.1 |  |  | 56445 | NaN | 0.0451 | No |
| 63 | ENSG00000179997 | AC010894.1 |  |  | 57169 | NaN | 0.0330 | No |
| 64 | ENSG00000226955 | AC104306.2 |  |  | 58542 | NaN | 0.0099 | No |
| 65 | ENSG00000237659 | RNASEH2CP1 |  |  | 58673 | NaN | 0.0078 | No |
Table: GSEA details [plain text format]

  

Fig 2: DNA\_REPLICATION(HSA03030)      
 Blue-Pink O' Gram in the Space of the Analyzed GeneSet

  

Fig 3: DNA\_REPLICATION(HSA03030): Random ES distribution      
 Gene set null distribution of ES for **DNA\_REPLICATION(HSA03030)**
